# The effects of *Cordyceps sinensis* phytoestrogen on estrogen deficiency-induced osteoporosis in Ovariectomized rats

**DOI:** 10.1186/1472-6882-14-484

**Published:** 2014-12-13

**Authors:** Da-wei Zhang, Zhen-lin Wang, Wei Qi, Guang-yue Zhao

**Affiliations:** Department of Orthopedics, Xi Jing Hospital, the Fourth Military Medical University, Xi’an, 710032 People’s Republic of China; The Surgery Department of 520th Hospital of PLA, Mian Yang, 621000 People’s Republic of China; Department of Orthopedic Surgery, the NO.113 hospital of PLA, Ningbo, 315000 People’s Republic of China

**Keywords:** *Cordyceps sinensis*, Isoflavones, Osteoporosis, Phytoestrogen, Estrogens

## Abstract

**Background:**

Isoflavones are naturally occurring plant chemicals belonging to the “phytoestrogen” class. The aim of the present study was to examine the effects of isoflavones obtained from *Cordyceps sinensis* (CSIF) on development of estrogen deficiency-induced osteoporosis in ovariectomized rats.

**Methods:**

After the rats were treated orally with CSIF, serum alkaline phosphatase (ALP), tartarate resistant acid phosphatase (TRAP), serum osteocalcin (OC), homocysteine (HCY), C-terminal crosslinked telopeptides of collagen type I (CTX), estradiol and interferonγ (IFN-γ) level were examined. At the same time, the urine calcium, plasma calcium, plasma phosphorus and the mass of uterus, thymus and body were also examined.

**Results:**

The beneficial effects of CSIF on improvement of osteoporosis in rats were attributable mainly to decrease ALP activity, TRAP activity, CTX level and IFN-γ level. At the same time, CSIF also increase the OC and estradiol level in ovariectomized osteopenic rats. The histological examination clearly showed that dietary CSIF can prevent bone loss caused by estrogen deficiency.

**Conclusion:**

The significant estrogenic activity of CSIF demonstrated that CSIF has significant estrogenic effects in OVX rats.

## Background

*Cordyceps sinensis* (CS) has been used as a tonic for longevity, endurance, and vitality for thousands of years by the Chinese [1]. Many studies have shown that CS modulates immune responses [2–4], inhibits tumor cell proliferation [5, 6], enhances hepatic function [7], regulates insulin sensitivity [8], decreases plasma cholesterol levels [9], and has hypotensive and vasorelaxant activity [10]. The effect of CS on osteoporosis had been studied in our former paper [11]. However, the metabolites that account for this effect have not been studies so far.

Many research groups have reported that isoflavones were associated with human health benefits such as decreased risks of various cancers, heart disease, cardiovascular disease, and increased antioxidative effects [12–14]. Isoflavones are naturally occurring plant chemicals belonging to the “phytoestrogen” class [15]. These compounds have structures similar to mammalian estrogens and display both estrogenic and anti-estrogenic effects [16].

Epidemiological studies indicate that the significant decrease in estrogen levels in women during the menopausal period causes osteoporosis, a major public health concern. Although hormone replacement therapy (HRT) can help to prevent and treat the menopausal syndromes, the side effects of HRT, such an increased risk of developing breast and endometrial cancer, prevent the acceptance of HRT [17, 18]. Now, there is an interest in using phytoestrogens to alleviate the menopausal symptoms, including development of osteoporosis. The aim of the present study was to examine the effects of isoflavones obtained from CS on development of estrogen deficiency-induced osteoporosis in ovariectomized rats.

## Methods

### Animals

Wistar rats (weighing 225 ± 25 g) were used in the study. This study was performed in accordance with the Guide for the Care and Use of Laboratory Animals. Care was taken to minimize discomfort, distress, and pain to the animals. The study was submitted to, and approved by, the Fourth Military Medical University institutional ethics committee.

### Isolation of CSIF

Cultured *Cordyceps sinensis* mycelium was obtained from Shandong HandongLukang Pharmaceutical Co., Ltd. (Shandong, China). The mycelium (100 g) was ground and extracted with 80% methanol. The methanol solution was evaporated to dryness and suspended in high-purity distilled water (500 mL) and then extracted using ethyl acetate (500 mL × 3). The extracts appeared as brown syrup (20 g) upon concentration. The sample was then fractionated using silica gel column chromatography in a stepwise gradient solvent system comprising 800 mL each of the chloroform/methanol mixtures. Fraction 5 was concentrated and loaded onto a Sephadex LH-20 column in a solution that contained a methanol/distilled water ratio of 8.5:1.5. Ten milliliter fractions were collected separately, and the final purification of compound was accomplished via HPLC using an isocratic aqueous acetonitrile solvent system. The CSIF was dissolved in CD3OD and analysed by 1DNMR and 2D NMR techniques as reported previously [19].

### Experimental design

The rats were randomly divided into five groups of animals, four ovariectomized (OVX) and another was given a sham-operation (control). Then group1 (sham) and 2 (OVX) were treated orally with 10-ml of saline, group 3, group 4 and group 5 were treated orally with CSIF (20 mg, 50 mg and 100 mg )for 8 weeks respectively. Body weight of the animals was recorded weekly.

On the last day of treatment urine was collected by micturation induced by manual pressure from overnight fasted animals and preserved at -20°C till further analysis [20]. At necropsy, blood was collected from dorsal aorta under ether anesthesia. After centrifugation, serum was harvested and kept at -20°C until analysis. Uteri were isolated. The absolute weight of uterine tissue was recorded and normalized with body weight (relative weight of uterus, i.e., weight of uterus per 100 g of body weight) of animals. The masses of thymus were also determined. The femoral neck was processed for mechanical testing. The left femur and L-4 vertebra bone were processed for mineral content and histological analysis.

### Urine calcium (Ca) content measurement

Calcium content of urine was measured by flame photometry (EHSY, China) after suitable dilution with double distilled water [21].

### Serum calcium and phosphorus measurement

Serum samples were analyzed for their calcium and phosphorus contents by the arsenazo-3 dye and ammonium molybdate colorimetric methods respectively.

### Serum hormone measurement

The serum was separated by centrifugation at 1000 × *g* for 10 min and then stored at -80°C until assay for E2, FSH, and LH. Serum E2, FSH, and LH concentrations were determined using radioimmunoassay (Sunbio, Inc., China).

### Plasma enzyme measurements

ALP and TRAP activity were determined by nitrophenol based method as described by Bessy et al. [22] and Godkar [23] respectively.

### Plasma proteins measurements

OCcontent was determined using an Osteocalcin EIA kit (Xinqidi bio -Technology, Inc., China) as described in the manufacturer’s directions. Two OC antibodies were employed, each directed toward the *N* or *C*-terminal OC molecule. HCY was measured by use of an enzymatic fluorescence polarization immunoassay on an Axsym analyzer (Abbott, Wiesbaden, Germany). CTX were quantified by ELISA (Sunbio, Inc., China).

### Solubility studies

The procedure for the preparation of bone powder was with the method described by Repo MA [24]. The mineral dissolution of bone powder was examined in acetate buffered solutions [25]. Briefly, 10 mg aliquots of powdered whole bone from rats were dispersed by sonication into 25 ml of 0.1 N acetate buffer pH = 5.0 at 37°C. Portions of the supernatant media that were freed of bone fragments by centrifugation for 2 minutes in an Eppendorf microfuge were harvested every 5 minutes for the first hour, hourly for the next 7 hours, and then at 24 hours. Calcium release from bone was followed by measuring the calcium content of the supernatant samples by atomic absorption.

### Representative images of the uterine sections

The reproductive organs (uterine) were carefully removed, cleaned, weighed and fixed in 10% formalin. Then it was stained with Hematoxylin and Eosin (H&E) and examined under light microscopy at 20× magnification.

### Histological analysis of lumbar vertebrae

The vertebrae were fixed in formaldehyde and then sectioned and stained with H&E. The bone structure was assessed under a light microscope.

### Data analysis

Group means were compared by Analysis of Variance (ANOVA) with GraphPad Prism (GraphPad Software, Inc.). Multiple comparison tests were performed with Tukey for significant differences at P < 0.05.

## Results

### Mass of uterus, thymus and body weights

**Figure 1:**
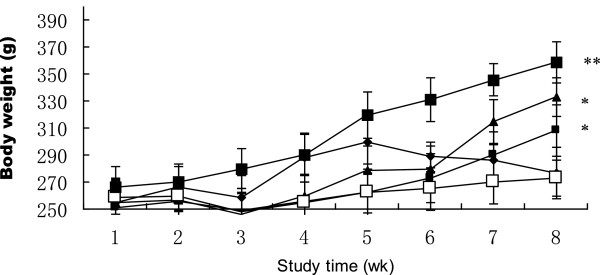
**Body weight changes for the study.** (◆sham group, □OVX group, ■CSIF-50group,▲CSIF-100 group and is ■CSIF-20 group ). CSIF-100 and CSIF-50 significantly increased body weights compared to OVX animals, Values are mean ± SEM. n = 10. *p < 0.05, ** *p* < 0.01compared to the OVX group at the same timepoint.

Absolute and relative uterine weight was significantly lower in OVX rats than in Sham rats. Significant increase was observed with CSIF (*p* < 0.05, *p* < 0.01) (Table 1). The OVX rats were shown by substantial increases in the thymus mass in relation to the sham rats (Table 1). CSIF significantly decreased the thymus mass of the OVX rats (*p* < 0.05, *p* < 0.01). The body weights of the rats are presented in Figure 1. Contrasted with the other groups, the body weights of rats in CSIF treated groups increased gradually 5 week later (*p* < 0.05 and *p* < 0.01).

**Table 1:** **Effects of CSIF on the mass of uterus and thymus in ovariectomized rats**

Groups	Thymus mass (mg)	Absolute weight of uterus (mg)	Relative weight of uterus (mg)
Sham	270.11 ± 23.10^**^	340.15 ± 20.10^**^	125.11 ± 11.03^**^
OVX	540.1 ± 31.11	79.21 ± 3.21	29.7 ± 8.6
CSIF-100	288.3 ± 20.00^**^	309.10 ± 11.20^**^	115.6 ± 31.0^*^
CSIF-50	333.0 ± 20.22^*^	266.23 ± 11.11^*^	84.0 ± 9.2^*^
CSIF-20	530.0 ± 18.20	100.30 ± 22.22	51.6 ± 8.8

Relative weight of uterus is the weight of uterus normalized with body weight (mg per 100 g of body weight). Values are mean ± SEM. n = 10. ^*^P <0.05 vs. OVX control; ^**^P <0.01 vs. OVX control.

### Urine calcium, serum calcium and serum phosphorus content

In the present study significant increase in urinary Ca excretion was observed in OVX control (Table 2). On the contrary, CSIF significantly decreased urinary Ca excretion (*p* < 0.01 and *p* < 0.05). The effects of treatment with CSIF on plasma total calcium and inorganic phosphate concentrations are shown in Table 2. All of treatments significantly decreased plasma total calcium and inorganic phosphate concentrations.

**Table 2:** **Effect of CSIF on serum calcium (Ca), inorganic phosphorus (P) and urinary calcium excretion in ovariectomized rats**

Different groups	Urinary Ca (mg/dl)	Serum Ca (mg/dl)	Serum P (mg/dl)
Sham group	48.61 ± 4.50^**^	8.00 ± 1.0^*^	5.10 ± 0.5 ^**^
OVX group	110.55 ± 12.2	15.93 ± 1.3	10.10 ± 0.4
CSIF-100	50.22 ± 8.55^**^	10.01 ± 0.4^*^	6.20 ± 0.2^**^
CSIF-50	71.22 ± 10.0^*^	11.10 ± 0.5^*^	7.99 ± 0.6^*^
CSIF-20	99.62 ± 12.1	13.88 ± 0 .4	8.88 ± 0.5

^*^P <0.05 vs. OVX control; ^**^P <0.01 vs. OVX control. (Mean ± standard deviation) (n = 10).

### Serum hormone measurement

Table 3 serum E2, FSH, and LH levels. The E2 levels of the OVX group were significantly decreased compared with those of the Sham group (*P* < 0.01). In contrast, the serum FSH and LH levels of the OVX group were significantly increased compared with those of the Sham group (*P* < 0.01). CSIF treatment restored serum E2, FSH, and LH to the same levels as in the Sham group.

**Table 3:** **Effect of CSIF on serum E**_**2**_ **, FSH, and LH levels in ovariectomized rats**

Different groups	E _2_((pg/mL))	FSH (mIU/mL)	LH (mIU/mL)
Sham group	28.4 ± 2.00^**^	1.80 ± 1.0^*^	1.10 ± 0.5 ^**^
OVX group	12.5 ± 2.20	4.30 ± 1.0	3.10 ± 0.4
CSIF-100	20.22 ± 2.50^**^	2.01 ± 0.4^*^	1.20 ± 0.2^**^
CSIF-50	16.00 ± 5.50^*^	3.10 ± 0.5^*^	1.99 ± 0.6^*^
CSIF-20	14.22 ± 2.10	3.88 ± 0 .4	2.80 ± 0.5

^*^P <0.05 vs. OVX control; ^**^P <0.01 vs. OVX control. (Mean ± standard deviation) (n = 10).

### Plasma enzyme measurements

In the present study significant increase in ALP and TRAP levels were observed in OVX control (Table 4). On the contrary, CSIF significantly decreased ALP and TRAP levels (*p* < 0.01).

**Table 4:** **Effects of CSIF on plasma enzymes in ovariectomized rats**

Groups	TRAP level (uM)	ALP level (mM)
Sham	0.22 ± 0.11^**^	3.25 ± 0.12^**^
OVX	0.82 ± 0.11	7.21 ± 0.10
CSIF-100	0.46 ± 0.03^**^	4.00 ± 0.03^*^
CSIF-50	0.55 ± 0.02	5.33 ± 0.06^*^
CSIF-20	0.75 ± 0.03	6.30 ± 0.02

Values are mean ± SEM. n = 10. ^*^P <0.05 vs. OVX control; ^**^P <0.01 vs. OVX control.

### Plasma proteins measurements

The effects of treatment with CSIF on OC level was shown in Table 5. All of treatments increased OC level. However, the values of CSIF-100 treated group were significantly higher than those of other treated group. Compared with OVX control, there were no significant differences in the increase of HCY content in CSIF groups (Table 5). The serum levels of CTX were significantly higher in the OVX group than in the other groups. The values of CTX-100 treated group were significantly higher than those of other groups (Table 5). The level of IFN-γ was significantly higher in the OVX group than that in the sham group. On the other hand, CSIF treated to the OVX rats decreased significantly the IFN-γ level (P < 0.05) (Table 5).

**Table 5:** **Effects of CSIF on plasma proteins**

Groups	Serum OC (ng/ml)	Serum HCY (μmol/L)	Serum CTX (ng/ml)	IFN-γ(ng/ml)
Sham	81.6 ± 5.2^*^	7.7 ± 1.1	75.6 ± 4.2^**^	0.82 ± 0.11^**^
OVX	56.4 ± 6.3^*^	9.9 ± 2.2	101.3 ± 5.1	2.70 ± 1.10
CSIF-100	81.1 ± 11.2	7.6 ± 1.1	80.2 ± 18.2^**^	1.60 ± 0.02^*^
CSIF-50	66.6 ± 14.0	8.1 ± 3.2	95.1 ± 19.3^*^	1.96 ± 1.2^*^
CSIF-20	59.6 ± 14.2	8.9 ± 2.0	99.3 ± 14.1^*^	2.55 ± 0.02

Values are mean ± SEM. n = 10. ^*^P <0.05 vs. OVX control; ^**^P <0.01 vs. OVX control.

### Solubility studies

**Figure 2:**
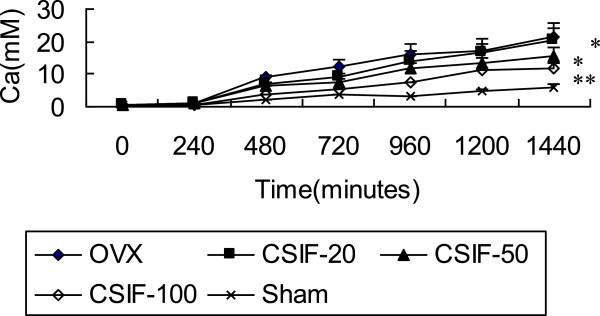
**Calcium solubility.** CSIF-100 and CSIF-50 significantly decreased the amount of calcium released after 24 hours compared to OVX animals, Values are mean ± SEM. n = 10. *p < 0.05, ** *p* < 0.01compared to the OVX group at the same timepoint.

As shown in Figure 2, mineral dissolution of bone from the CSIF treated rats were reduced as compared with the OVX group over the entire time period measured. The differences between them and OVX group in the rate of dissolution as well as the amount of calcium released at 24 hours were highly significant (P < 0.01).

### Effects of CSIF on the uterine sections

**Figure 3:**
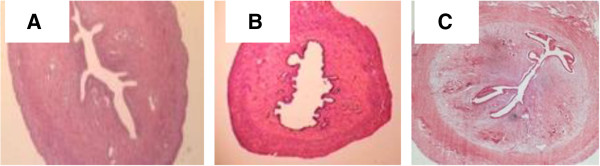
**Representative images of the uterine sections.** Representative images of the uterine sections from OVX SD rats treated with CSIF-100. **(A)** sham group, **(B)** OVX group, **(C)** CSIF-100 group. There was an obvious increase in the size of the lumen following CSIF-100 treatment. Sections were stained with H&E, Magnification X4.

Under light microscopy, we observed marked atrophy of the rat uterus to about half its former size once the rats are ovariectomised (OVX group). The size increased to near normal in CSIF-treated rats (Figure 3C). Likewise the endometrial thickness showed atrophy in the OVX group (Figure 3B). After 8 weeks administration of CSIF at all doses caused a reversal of the vaginal atrophy. This effect was accompanied by mild hyperplasia of vaginal epithelium. Cytoplasmic vacuolization of vaginal epithelium was noted in rats on high dose of 100 mg CSIF (Figure 3C). The general histological features of the squamous epithelium of the vagina of the CSIF100-treated rats closely resembled to that in SHAM rats (Figure 3A).

### Effects of CSIF on bone structure changes

**Figure 4:**
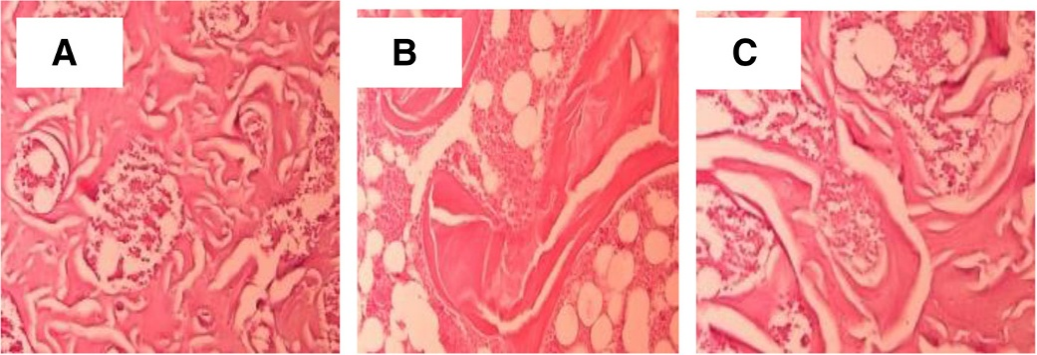
**Histology of lumbar vertebrae.** The bone structure was photographed under a light microscope. It shows that there was a significant trabecular bone loss in the OVX rat **(B)**, whereas the CSIF-100 treatment rat section **(C)** seems near normal compared with sham-operated animals **(A)**.

A sample photomicrograph is presented in Figure 4; it is quite clear that trabecular bone loss is much higher in the vertebrae of rat with OVX (Figure 4B), whereas the vertebrae of CSIF100-fed OVX rat appear to be near normal (Figure 4C).

## Discussion

Phytoestrogens are a diverse class of non-steroidal compounds that have an affinity for estrogen receptors α and β for the peroxisome proliferator-activated receptor (PPAR) family and for the aryl hydrocarbon receptor [26–28]. Many phytoestrogens counteract the cellular derailments that are responsible for the development of metabolic syndrome [29–31]. Here we propose the effects of isoflavones, which are naturally occurring plant chemicals belonging to the “phytoestrogen” class, obtained from *Cordyceps sinensis* on development of estrogen deficiency-induced osteoporosis in ovariectomized rats.

According to ancient descriptions, *Cordyceps sinensis* possesses important pharmacological activities for protecting lung and kidney functions and in nourishing essential and vital energy [32]. Body weight is a general index of the health of an animal. The increases in body weight by CSIF may contribute to maintaining the elevated periosteal bone formation and inhibit endosteal bone resorption [33].

Ovariectomized (OVX) animal models, in a variety of species, have been used to evaluate the mechanism of or to assess the effect of drugs in estrogen deficiency. The ovariectomized rats were shown by substantial increases in the thymus mass in relation to the sham-operated control rats (Table 1). The hypertrophy of the thymus resulting from ovariectomy was inhibited by CSIF. The results indicate CSIF could enhance the immune functions in OVX animals.

Uterus is a primary target organ for estrogen. The uterotrophic assay is a standard tool for determining the estrogenic activity of a given substance in vivo [34]. This study has shown for the first time that the oral administration of CSIF has significant estrogenic effects in OVX rats, including dose-dependently increasing uterine weight, restoring the uterine structure. In the current study, absolute and relative uterine weight was significantly lower in OVX rats than in Sham rats. Significant increase was observed with CSIF in weight of atrophic uterus (Table 1). These results may account for the possible mechanism of the action of CSIF.

It has been reported that the serum biochemical parameters, such as Ca and P are normal in osteoporosis in man [35]. However, in the present investigation, significant differences in Ca and P levels were found in different group. CSIF treatments significantly decreased plasma total calcium and inorganic phosphate concentrations. At the same time, CSIF significantly decreased urinary Ca excretion. It agrees with the above results of the increases in body weight by CSIF and consistent with the finding that body weight may contribute to maintaining the elevated periosteal bone formation and inhibit endosteal bone resorption [33].

It has been reported that phytoestrogens have the beneficial effect of restoring the profile of such sex hormones as E2, FSH and LH in women. FSH and LH are key stimulators for follicular development. The preovulation secretion of FSH and LH is negatively regulated by circulating E2 via the feedback control system of the hypothalamic-pituitary-ovarian axis [36].

In the current study, CSIF significantly increased serum E2 levels and attenuated the elevated serum LH and FSH levels resulting from the removal of E_2_ in OVX rats, indicating that CSIF has a beneficial role in regulating hypothalamic-pituitary function.

ALP is a non-collagenous protein secreted by osteoblast, which is essential for bone mineralization [37]. Increased ALP level in serum has been observed in conditions such as rapid bone loss [38] and fracture risk [39, 40]. TRAPis secreted by osteoclasts during bone resorption, and serum TRAP activity correlates with resorptive activity in disorders of bone metabolism. In the present study, CSIF significantly decreased ALP and TRAP levels commonly used bone remodeling markers. It suggested that the potency of CSIF is due to decrease ALP activity, TRAP activity in OVX rats.

Osteocalcin (OC) is known as serum markers reflecting osteoblast activities including bone formation and turnover [41]. Treatment with CSIF increased OC level. The result suggested that the treatment with CSIF induce the secretion of OC after oral administration. Bone consists of a calcified organic matrix, which is composed of 90% type I collagen [42]. Higher C-terminal crosslinked telopeptides of collagen type I (CTX) levels are associated with lower bone mineral density values in osteoporosis [43]. The serum levels of CTX were significantly lower in the CSIF group than in the other groups. Substantial evidence demonstrates that have shown that IFN-γ enhances osteoclast generation in cultures of peripheral blood from osteopetrotic patients [44]. Our study was consistent with this finding. However, CSIF treated to the OVX rats decreased significantly the IFN-γ level (P < 0.05).

These findings suggested that the effect of CSIF on osteoporosis is due to decrease CTX and IFN-γ level, as well as increase the OC level in plasma in OVX animals.

The mineral dissolution of bone from the CSIF treated rats were reduced as compared with the control group over the entire time period measured. The result assumed that the decreased solubility of treated bone accounts in part for its decreased resorbability. It is consistent with the findings of above results. These findings suggested that the potency of CSIF is due to dual effects on both decreased resorption and increased formation. The histological examination clearly showed that dietary CSIF can prevent bone loss caused by estrogen deficiency.

## Conclusions

This study has shown for the first time that the oral administration of CSIF has significant estrogenic effects in OVX rats, including dose-dependently increasing uterine weight, restoring the circulating E2, FSH, LH, ALP, TRAP, OC, CTX and IFN-γ levels, and preventing bone loss due to E2 deficiency. The significant estrogenic activity of CSIF demonstrated that CSIF has significant estrogenic effects in OVX rats.

## Authors’ information

Da-wei Zhang and Zhen-lin Wang are co-first authors.
